# Biological Machine Learning Combined with *Campylobacter* Population Genomics Reveals Virulence Gene Allelic Variants Cause Disease

**DOI:** 10.3390/microorganisms8040549

**Published:** 2020-04-10

**Authors:** DJ Darwin R. Bandoy, Bart C. Weimer

**Affiliations:** 1 100 K Pathogen Genome Project, Department of Population Health and Reproduction, School of Veterinary Medicine, University of California Davis, Davis, CA 95616, USA; 2 Department of Veterinary, Paraclinical Sciences, College of Veterinary Medicine, University of the Philippines Los Baños, Los Baños 4031, Philippines; drbandoy@ucdavis.edu

**Keywords:** *porA*, infectious disease, XGBoost, *Campylobacter*, abortion, protein modeling, artificial intelligence, allelic variation, bacterial metastasis

## Abstract

Highly dimensional data generated from bacterial whole-genome sequencing is providing an unprecedented scale of information that requires an appropriate statistical analysis framework to infer biological function from populations of genomes. The application of genome-wide association study (GWAS) methods is an appropriate framework for bacterial population genome analysis that yields a list of candidate genes associated with a phenotype, but it provides an unranked measure of importance. Here, we validated a novel framework to define infection mechanism using the combination of GWAS, machine learning, and bacterial population genomics that ranked allelic variants that accurately identified disease. This approach parsed a dataset of 1.2 million single nucleotide polymorphisms (SNPs) and indels that resulted in an importance ranked list of associated alleles of *porA* in *Campylobacter jejuni* using spatiotemporal analysis over 30 years. We validated this approach using previously proven laboratory experimental alleles from an in vivo guinea pig abortion model. This framework, termed μPathML, defined intestinal and extraintestinal groups that have differential allelic *porA* variants that cause abortion. Divergent variants containing indels that defeated automated annotation were rescued using biological context and knowledge that resulted in defining rare, divergent variants that were maintained in the population over two continents and 30 years. This study defines the capability of machine learning coupled with GWAS and population genomics to simultaneously identify and rank alleles to define their role in infectious disease mechanisms.

## 1. Introduction

Comparative microbial genomics has relied on pangenome comparisons to characterize changes in the core and flexible genome, which provided genes lists associated with gene changes but had little association with determine the causal genes for a disease [1]. An alternative approach to this perspective is the use of genome-wide association (GWAS) analyses, which are commonly used in mammalian genomics, in an effort to refine the estimates of specific genes of interest for microbial gene association with phenotype, such as pathogenicity. However, a limitation of GWAS is the sequential examination of single loci, which prevents simultaneous analysis of multiple genes or allelic variants that may interact to cause a phenotype. This is a severe limitation in comparative bacterial genomics, especially as the population of bacterial genomes continues to grow reaching nearly 1 million in With this large number of genomes that often represent multiple genomes for a single species or serotype, it is appropriate to treat each genome as a member of a population of individuals that are spatiotemporally distributed. A spatiotemporal analysis framework makes it possible to examine the non-linear evolutionary rates of each genome in combination with specific selective conditions for all of the alleles of specific genes found in very large populations that are maintained or diluted in populations that are consistently associated with microbial phenotypes—especially disease and tissue tropism. However, this approach adds to an ever-growing big data problem for microbial genomics requiring new approaches for microbial comparative genomics and statistical methods. To address these limitations with highly dimensional bacterial genome analyses, we can use the integration of multidimensional metadata alongside the strain differences to create a robust analysis framework that can be used with GWAS [2].

A compounding limitation of this framework is the lack of appropriate statistical models that underpin this approach in bacteria since it is unknown when the populations are normally distributed or evolving non-linearly. As with all big data problem sets, the multiple comparisons problem requires a correction, such as the Bonferroni correction, to adjust the *p*-value, which moves this correction to problems that are beyond what was contemplated when this approach was invented for gene expression (Table 1) [3]. Further, the assumption that each gene or allele behaves independently within the genome is conceptually flawed in bacteria considering the operon configuration, horizontal gene transfer (e.g., plasmids), and the evolution rate of bacteria are on the order of minutes rather than years in mammals. Hence, alternative analyses that are biological and statistically compatible need to be defined for bacterial population genomics.

Coupling GWAS, population microbial genomics, and machine learning is poised to be a robust alternative to classical GWAS or pangenome comparisons alone; however, the combination of these methods will likely simultaneously discover changes in microbial genomes and gene variation that span the spatiotemporal scale, genome plasticity, and large numbers of selection conditions that result in gene variation that maybe causal in disease but with only a subset of gene variants or specific alleles with cause variation in the disease symptoms. Moreover, this combination (coined here as μPathML) produces a statistically valid method that results in biologically informative rankings for each genome, gene, and allele that are not determined from any of the individual analyses alone. These advantages, combined with downstream inspection of the prioritized rankings, further power biological discovery to bring insightful observations about the genome and the phenotype, especially when large genome populations are used in the analysis, from very divergent populations of alleles. To extend this concept, highly divergent sequences with similar function that are missed with automated gene calling approaches can be brought back into biological relevance, especially if gene mutations are tracked as new genes as was done by Weimer et al. [4] and Kaufman et al. [5,6,7].

An analytical strength of machine learning for use in microbiology is the ability to define functional relationships from population-scale genome comparisons or genes without a priori definition of the underlying mechanism of change or specific phenotype limitations [8]. This distinctive advantage makes machine learning superior to classical statistical tests for microbial applications because the individual genomes are so highly variable in gene content and phenotypes that lead to varying displays of the disease and tissue location [9,10]. This is particularly useful in bacteria when causal genes act in combination or do not evolve linearly, gene variants interact, varying evolutionary rates between genes within the same genome, or assumptions of normal distribution are violated in part due to the selection conditions [2,11]. These biological conditions and parameters are incompatible with the assumptions of linear or correlative statistics, which is compounded with data reduction methods, which provide a very small snapshot of the genome variation that yield associations with low predictive value that is compounded by highly variable genomes of the same species [12,13,14,15], such as with microorganisms.

## 2. Materials and Methods

### 2.1. Biological Feature Engineering

Biological feature engineering entails selection of pertinent controls and cases for μPathML analysis. The genomes between gastrointestinal and extraintestinal abortive isolates. *Campylobacter jejuni* controls were downloaded from Patric 3.5.28 (https://www.patricbrc.org/), 1 June 2019 (Table S2). Abortive extraintestinal genomes of *C. jejuni* were obtained from the Sequence Read Archive (SRA; Table S3) [16]. Fastq files were assembled using Shovill (version 1.0.4; https://github.com/tseemann/shovill). Assembled files were annotated with Prokka (version 1.13.3) [17]. Variant calling was done with the reference sequence *C. jejuni* NTC11168 with Snippy (version 4.3.5; https://github.com/tseemann/snippy) as previously described [18].

### 2.2. Gradient Tree Boosting as GWAS Framework

GWAS variants generated from the biological feature engineering step were used as input for XGBoost. The original source code for implementing gradient tree boosting is available at https://xgboost.readthedocs.io/. A confusion matrix was generated and used to assess the performance of the model (Table S4). The relative importance of the predictive model was used as the GWAS hits.

### 2.3. Tetris Plot

Classical GWAS hits are displayed as the negative logarithm of the *p*-value in Manhattan plots, hence we formulated a novel visualization of the ranked alleles generated by the machine learning model to highlight the difference between approaches—we call this GWAS hit visualization a Tetris plot and used when color coding the relative importance values of the associated alleles derived from the XGBoost (green being associated and red being non-associated). The source genome is plotted on the *y*-axis and genomic coordinates on the *x*-axis overlaid with a GWAS hits presence or absence matrix.

### 2.4. Population-Wide Whole-Genome Phylogeny

The genome distance metric was calculated using genome-wide k-mer signatures to generate the population-wide phylogeny with a k-mer size of 31 scaled to 1000 with Sourmash [19]. The resulting genome-wide k-mer distance was visualized as an all-against-all heatmap [19].

### 2.5. Protein Modeling

Assembled genomes were annotated using Prokka (Version 1.13.3) and PorA protein sequences were extracted for protein modeling using Swiss Model [20,21]. The most homologous protein was used as template for protein modeling. Illustrate (https://ccsb.scripps.edu/illustrate/) was used to generate the protein visualization of the alleles. Ranked BioML alleles identified by visual inspection of the Tetris plot, via the ranked variable importance were used to inspect the protein structures.

## 3. Results

**Figure 1:**
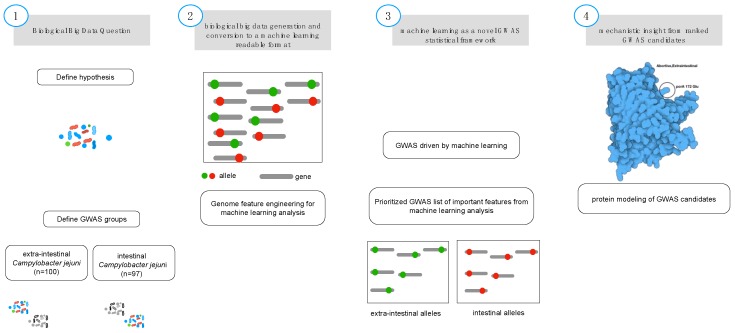
Biological feature engineering of genomic data for machine learning analysis. A critical step in feature engineering is selection of the appropriate comparison groups to enable classification of alleles that are related to the specific phenotype of interest (i.e., intestinal (controls; diarrheal; *n* = 108) and extraintestinal (cases; abortive; *n* = 85) (Step 1). Population-wide allelic variants (red dot = intestinal, green dot = extraintestinal) that result from variant calling (Step 2) and are used as the input features for machine learning analysis (Step 3). The predicted model generated from the machine learning analysis is inspected for the most predictive features using biological context, input, and protein modeling (Step 4) that represents a non-synonymous mutation from the genomic population of allelic variants (*n* = 193).

**Figure 2:**
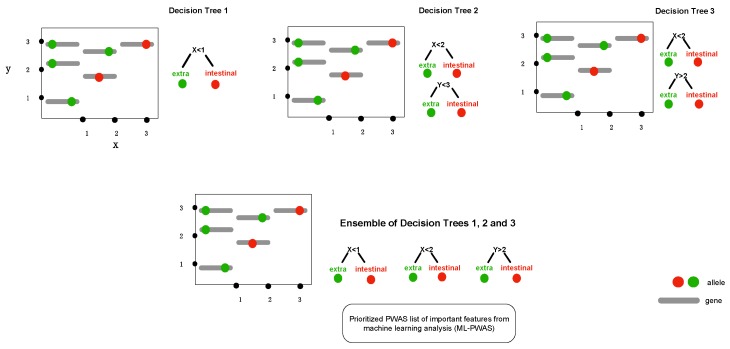
The conceptual framework diagram depicting machine learning in bacterial genome-wide association using extreme gradient boosting (XGBoost). Boosting is a technique of combining a set of weak classifiers or decision trees to increase prediction accuracy. Red dots represent an allelic variant, each grey bar represents a unique allele. Individual decision trees (1, 2, 3) fail to fully capture the allelic variants associated with the phenotype (e.g., extraintestinal abortion), but combining the trees together results in a process called boosting as it increases the discriminative power.

In this study, we coupled GWAS with machine learning and population bacterial genomics (Figure 1) creating a broadly applicable framework that was validated using previously published verified alleles of a virulence gene that causes abortion in livestock [16,22,23]. We hypothesized that specific alleles of a single gene (i.e., *porA*) define the ability of *Campylobacter* for extraintestinal invasion and further are causative of abortion with specific alleles. This was done using a wet-lab validated data set containing 100 genomes [16,22,23] combined with machine learning using extreme gradient boosting (XGBoost) [24,25]. The ability to interrogate the predictive features emerged as a tool to determine mechanistic function in complex biological systems [26]. XGBoost implements adaptive optimization within the functional space by iteration of the weak learners into strong learners represented by decision trees, where each new decision tree is generated by factoring the residuals generated from the difference from the observed to the predicted feature (Figure 2; Table S1).

We used previously validated wet-lab data with a tetracycline-resistant strain of *C. jejuni* causing abortion in sheep [16,22,23] as the validation training set for μPathML analysis. Previously, these robust studies used a pairwise comparison to identify 8000 single nucleotide differences (SNP) differences between a reference genome and an abortive strain that subsequently utilized genetically transformed genomes to identify specific allelic variants that cause abortion in a model system. We validated μPathML using 85 genomes that span 30 years and multiple locations as a reference set of cases and 108 control genomes of intestinal and diarrheal isolates. This approach permitted exploration of the bacterial population genomic space of this organism by linking different phenotypes to validated genome variation (Figure 1). Biological feature engineering of this collection identified 1.2 million SNPs, which is not tractable using in vivo infection studies, to determine the role of all SNPs in this gene across the bacterial population to cause this disease. To use this approach at a big-data scale, we hypothesized that genomic changes evolved in gastrointestinal *C. jejuni* resulting in an abortive phenotype; hence, invading the intestine and progressing to other tissues—in this case the placenta resulting in abortion. Applying μPathML analysis to the population of gastrointestinal, diarrheal *C. jejuni* versus extraintestinal, abortive phenotypes produced a ranked set of alleles in a ranked order of importance to the phenotype (i.e., abortion) (Table S1).

**Figure 3:**
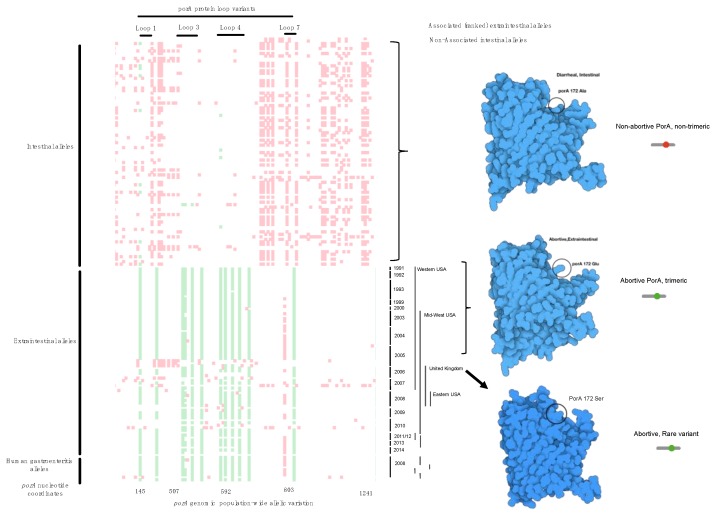
Comparative plot of SNP loci along the *porA* gene in all genomes. We termed this a Tetris plot as an alternative visualization of genome-wide association hits because they are ranked and display only the loci that vary to produce a non-synonymous mutation. The *y*-axis contains individual genomes from the cases and the controls, while the *x*-axis contains the GWAS SNP loci (green), the non-disease-associated SNPs (red), open space (white) are loci that are identical in the gene sequence. Temporal and geographic metadata on the right side of the Tetris plot provides context for mutational enrichment over 30 years and multiple distant locations in North America and the UK. The enriched SNP variation produced different protein structures (far right in blue) as the corresponding protein model by location within the animal by SNP. Protein structural features corresponding to the ranked GWAS variants are annotated on top, and below the plot are the nucleotide coordinates. Rare variants (homology <75%) were not included by the variant caller in this visualization, but manual inspection provided a method to find these variants.

μPathML analysis identified 14 *porA* loci as the most important alleles ranging from 1.0 to 0.65 scaled importance out of the 1.2 million SNPs (Table S1). These ranked loci were compared by body location (Figure 3), which further clarified the location of these SNPs and indels that simultaneously presented the ranked associated allelic variants within the phenotype of interest as detected with μPathML as well as the non-associated alleles. This analysis method enabled modeling of various protein structures for PorA between abundant versions to hybrid variation and rare variants that were not captured by automated gene calling, machine learning alone, or classical statistical testing. Regions within *porA* from the cases expressing different allelic versions were further explored for each genome and ranked *porA* allele to determine the implication for biological function important in the disease. Protein structures were modeled to examine the changes in protein configuration, initially yielding four distinct groups (Figure 3) that ranged from non-abortive to variations of proteins all of which caused abortion. These alleles were directly compared to those validated in vivo and found to be linked to specific protein loops within alleles verified previously [16,22,23]—in all cases μPathML found each of those to be biologically important for abortion and found new hybrid versions of the protein that were previously unrecognized.

**Figure 4:**
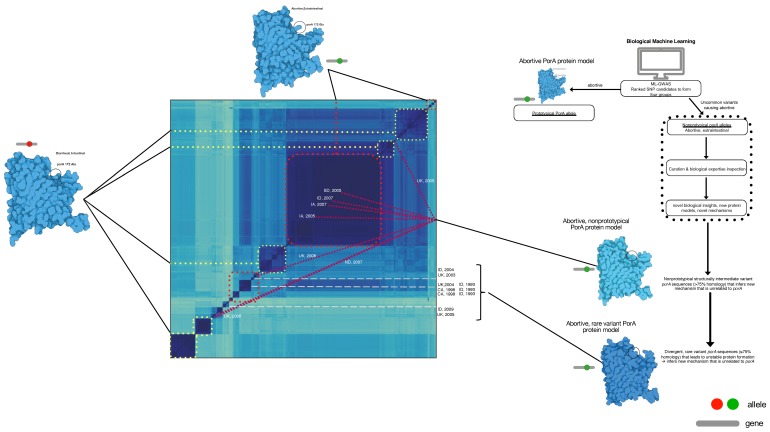
Whole-genome distance matrix using MinHash depicting an all-against-all comparison of genome diversity for all isolates used in this study overlaid with the *porA* variant associated with body location and disease phenotype. Genotypes and *porA* alleles are connected in this depiction to examine the association between intestinal/diarrheal location (yellow dot boxes), prototypical extraintestinal/abortive (red dot boxes), non-prototypical *porA* alleles in extraintestinal/abortive (maroon lines), and rare *porA* variants in extraintestinal/abortive (grey dashed lines) were co-located to their respective genomes in the genotype map. For the non-prototypical variants, the year and location of isolation was included to depict the variation over time and space in the maintenance of a minority population of *porA* alleles of extraintestinal abortive *C. jejuni*. The diagram to the right depicts the process used for this analysis.

Further verification of the approach found that each of the top-ranked alleles were located in loops 1, 3, 4, 7, which perfectly verifies the published observations using genetic manipulation and a model infection [16,22,23]. By examining every genome from abortion cases, we found variants that were between 90% identical with >75% protein homology that were designated as non-prototypical variants because the sequence variation was high enough to change protein structures but within the parameters used for automated gene calling. In a limited set of alleles, the *porA* gene was so divergent that they were missed using automated gene calling but were recovered with manual curation of the μPathML output. Recovery of these genes created a third group of rare variant alleles that also caused abortion (Figure 4; protein homology <75%). This result provides a foundation for functional variation of a core gene from all *Campylobacter* and further provides insight into the variability of *porA* as a virulence factor, even in highly divergent alleles.

All of the variants were mapped to the whole-genome phylogeny to determine whether the alleles were co-evolving at the same rate with the genome population (Figure 4). While some of the alleles were associated with similar genomes, most of the alleles were found in >2 strain genotypes. Prototypical allelic variants clustered in the largest genomic group of abortive isolates, as did some of the non-prototypical *porA* variants. However, there was significant genome variation among the population where the *porA* alleles were distributed among the genome population. Rare *porA* variants were distributed within different genomic groups and over a 15-year span between North America and the UK. The extensive allelic variation of *porA*, as well as the different genotypes, indicates that a genome surveillance system based on SNPs or a small number of genes would result in false negatives, and attempts to link these genomes to an outbreak would be unsuccessful. In combination, these observations indicate that μPathML produced a ranked list of biologically important alleles that were validated with those that were previously shown to be causal in abortion for the exact SNP and the protein loop location. Together, these observations verified that μPathML was capable of accurately identifying the exact SNPs in *porA* that cause abortion.

## 4. Discussion

**Figure 5:**
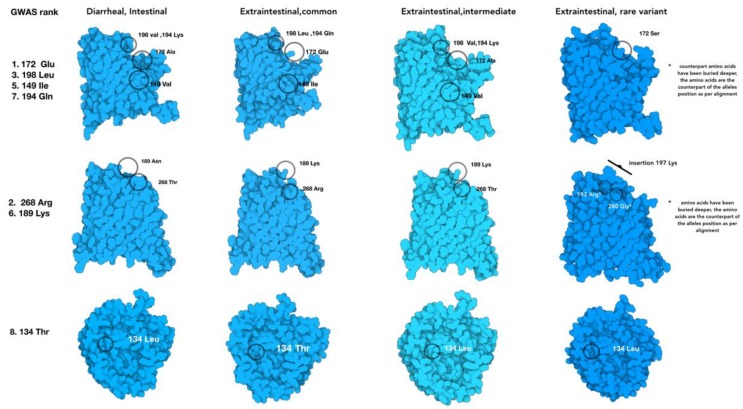
Protein models of the four groups of *porA* allelic variants that change the PorA protein model structure relative to the isolate location in the host and the disease outcome. The amino acids corresponding with the BioML top-ranked alleles are labeled in the common variant of PorA, while the rest show the substituted amino acid in their respective position.

Since each μPathML allele was accurately validated for accuracy to empirical studies based on animal models and genetic evidence, we broadened the examination of the protein changes from the ranked alleles to determine whether the protein structure variation contained a specific feature or amino acid substitution that was linked to abortion (Figure 5). The first six top-ranked alleles contained various amino acid substitutions for each *porA* sequence and multiple PorA protein models. However, Lys_189_ was conserved among the extraintestinal *porA* allele and Asn_189_ was found in the intestinal alleles. Lys mutation changes are the most impactful in membrane pore structure and are one of the tenets of membrane topology as the positive inside rule [27,28]. The positive inside rule describes the observation across membrane pores that associate positively charged amino acids within the cytoplasm and negatively charged amino acids in the extracellular domain. Membrane topology can radically change from being oriented inside the membrane (exposed to the periplasm in this case) to outside the membrane with a single Lys_189_ mutation, suggesting that this mutation may flip the protein orientation in the membrane relative to AsnWithin the adjacent protein structure, Lys snorkeling effectively minimized the non-polar chain component by burying it in the hydrophobic domain and at the same time exposed the polar component to the aqueous domain, another single amino acid change that alters the topology of the membrane domain [29]. Bacterial membrane pore flipping could be a potential mechanism to avoid recognition by the immune system and enhance of ion transport for bacterial metabolism. In atypical (i.e., hypervariable alleles) this position is buried in a deeper position due to insertional mutation in rare variants, the inserted amino acids contain Lys_197_, a new mutational position as compared to the prototypical protein model. Additionally, insertions in the rare variants reduce the homology to <75% leading to more extensive protein structural changes, as expected, that changed the PorA protein orientation in the membrane and retained the abortive phenotype. This situation is troublesome for traditional homology approaches and would be missed completely using comparative genomics alone. However, μPathML combined with biological tracing effectively identified this situation to successfully link multiple genotypes, protein structural models, and the disease to provide a validated basis that presents multiple underlying mechanisms for the abortive phenotype. Importantly, this finding highlights the need to examine genes and their alleles to determine causality. In this case, the role of *porA* has been controversial [30] in causing disease, and it is likely linked to the specific allele that is present and not just to the gene.

## 5. Conclusions

This study utilized a combination of GWAS, population bacterial genomics, and machine learning to identify and rank allelic variants that correspond to biologically validated alleles of *porA* that cause abortion. The μPathML analysis was further supported by the longitudinal and spatial conservation of the *porA* gene coupled to protein substitutions that led to important and biologically relevant changes in the structure to change activity that was linked to allelic variation conserved over 30 years and multiple global locations. A Tetris-plot visualization provided an avenue to discover divergent and rare variants that provided further insight with protein modeling that uncovered protein substitutions resulting in localization changes that affect activity and isolation localization in the host. Together these results demonstrate and validate a novel method, μPathML, to discover biological variation combined with established mechanisms using population bacterial genomics. This approach provides an avenue to leverage the massive amount of bacterial genomic sequences to uncover new mechanisms of disease with potential to provide therapeutic approaches.

## Figures and Tables

**Table 1:** Exemplar comparison of statistical metrics of genome-wide association study (GWAS) versus machine learning metrics. Allelic variant association with phenotype using XGBoost. An allele can be very large, ~8000 for *porA* for a pairwise comparison. Using a population of this gene from 200 genomes created a population variation of 1.2 million variants that can be ranked with an estimation of importance to association with the disease phenotype, abortion in this case.

	GWAS Statistical Metrics		Machine Learning Coupled to GWAS Metrics	
Allele	GWAS *p*-Value	Bonferroni Corrected *p*-Value	Candidate Ranking	Feature Importance
X_1_	0.001	8.3 × 10^−10^	1	80
X_2_	0.001	8.3 × 10^−10^	2	75
X_3_	0.001	8.3 × 10^−10^	3	70
X_n_	0.001	8.3 × 10^−10^	Rank_n_	Importance_n_

## Supplementary Materials

The following are available online at https://www.mdpi.com/2076-2607/8/4/549/s1, Table SRanked allelic variants using BioML. Table SMetadata for extraintestinal *Campylobacter jejuni.* Table SMetadata for intestinal *Campylobacter jejuni.* Table SConfusion matrix and derived model metrics for the XGBoost model with extraintestinal *Campylobacter jejuni*. TP = true positive, FN = false negative, FP = false positive, FN = false negative.
