# Supplementary material for: Biological Machine Learning Combined with Campylobacter Population Genomics Reveals Virulence Gene Allelic Variants Cause Disease
Source: Microorganisms. 2020 Apr 10;8(4):549. doi: 10.3390/microorganisms8040549 (PMC7232492; doi:10.3390/microorganisms8040549)
Supplement: Supplementary file 1 [file microorganisms-08-00549-s001.pdf]

Table S1. Ranked allelic variants using BioML.

Rank	allele	relative importance	scaled importance	percentage
1	porA_507/1275	89.41	1	0.0048
2	porA_513/1275	89.39	0.9998	0.0048
3	porA_1099/1275	86.78	0.9706	0.0046
4	porA_651/1275	86.28	0.965	0.0046
5	porA_803/1275	86.02	0.9621	0.0046
6	porA_394/1275	85.31	0.9542	0.0045
7	porA_592/1275	84.53	0.9455	0.0045
8	porA_548/1275	83.56	0.9346	0.0044
9	porA_445/1275	83.19	0.9304	0.0044
10	proS_1373/1707	83.12	0.9297	0.0044
11	porA_562/1275	82.20	0.9194	0.0044
12	porA_435/1275	82.00	0.9171	0.0044
13	porA_372/1275	81.52	0.9117	0.0043
14	porA_580/1275	81.20	0.9082	0.0043
15	porA_400/1275	81.16	0.9077	0.0043
16	porA_250/1275	79.46	0.8887	0.0042
17	porA_459/1275	79.24	0.8863	0.0042
18	ubiE_2_255/774	78.06	0.8731	0.0042
19	kpsM_157/783	77.53	0.8672	0.0041
20	hemL_762/1275	77.09	0.8623	0.0041
21	proS_1512/1707	76.92	0.8603	0.0041
22	sdhA_1599/1836	76.42	0.8548	0.0041
23	ackA_1114/1191	76.41	0.8547	0.0041
24	kpsD_1065/1659	76.37	0.8542	0.0041
25	porA_411/1275	75.90	0.8489	0.004
26	tatC_94/738	75.65	0.8461	0.004
27	proS_67/1707	75.64	0.846	0.004
28	nrdA_315/2370	75.14	0.8405	0.004
29	kpsD_55/1659	75.04	0.8393	0.004
30	ubiD_1006/1803	74.66	0.8351	0.004
31	nrdA_333/2370	74.22	0.8302	0.004
32	dnaJ_318/1122	74.21	0.8301	0.0039
33	hisG_453/900	73.89	0.8264	0.0039
34	proS_1614/1707	73.59	0.823	0.0039
35	proS_1594/1707	73.58	0.8229	0.0039
36	proS_1364/1707	72.98	0.8163	0.0039
37	pnp_1347/2160	72.97	0.8162	0.0039
38	dnaJ_950/1122	72.97	0.8161	0.0039

39	purB_1071/1329	72.89	0.8153	0.0039
40	nadE_653/741	72.89	0.8152	0.0039
41	ppiB_288/483	72.56	0.8115	0.0039
42	ispB_118/894	72.35	0.8093	0.0039
43	purB_1304/1329	72.28	0.8085	0.0038
44	purB_1167/1329	72.27	0.8084	0.0038
45	pnp_930/2160	72.26	0.8083	0.0038
46	pheT_268/2322	71.34	0.7979	0.0038
47	pheT_218/2322	71.33	0.7978	0.0038
48	ruvC_342/555	71.32	0.7977	0.0038
49	ruvC_324/555	71.02	0.7943	0.0038
50	mdtJ_198/342	69.96	0.7825	0.0037
51	cfiB_194/1446	69.95	0.7824	0.0037
52	pseG_341/825	69.95	0.7823	0.0037
53	ttcA_621/756	69.24	0.7744	0.0037
54	ahpC_397/597	68.39	0.765	0.0036
55	ackA_165/1191	68.25	0.7634	0.0036
56	arsB_327/1287	68.02	0.7608	0.0036
57	dnaJ_522/1122	68.01	0.7607	0.0036
58	purQ_425/672	67.80	0.7583	0.0036
59	ttcA_715/756	67.63	0.7564	0.0036
60	kpsD_498/1659	67.62	0.7563	0.0036
61	porA_642/1275	67.59	0.756	0.0036
62	ahpC_444/597	67.24	0.752	0.0036
63	porA_541/1275	67.22	0.7518	0.0036
64	rimO_629/1320	66.53	0.7442	0.0035
65	rimO_429/1320	66.52	0.7441	0.0035
66	flaA_1_1581/1719	66.51	0.744	0.0035
67	mdtJ_280/342	66.42	0.7429	0.0035
68	nth_2_165/687	66.24	0.7409	0.0035
69	glgC_2_590/762	66.09	0.7392	0.0035
70	arsB_726/1287	66.02	0.7384	0.0035
71	arsB_661/1287	65.96	0.7378	0.0035
72	arsB_636/1287	65.96	0.7377	0.0035
73	arsB_608/1287	65.95	0.7376	0.0035
74	fumC_657/1392	65.75	0.7355	0.0035
75	nrdA_1317/2370	65.60	0.7337	0.0035
76	glgC_2_372/762	65.28	0.7302	0.0035
77	ruvC_337/555	65.28	0.7301	0.0035
78	pcaB_78/1368	65.11	0.7283	0.0035

79	kdsD_294/948	65.08	0.7279	0.0035
80	ahpC_558/597	64.67	0.7233	0.0034
81	ahpC_264/597	64.66	0.7232	0.0034
82	nrdA_1206/2370	64.48	0.7212	0.0034
83	parB_429/837	64.45	0.7208	0.0034
84	proC_652/732	64.44	0.7208	0.0034
85	parB_414/837	64.44	0.7207	0.0034
86	parB_333/837	64.43	0.7207	0.0034
87	mnmG_1578/1860	64.42	0.7206	0.0034
88	cfiB_954/1446	64.41	0.7205	0.0034
89	arsB_291/1287	64.40	0.7203	0.0034
90	arsB_137/1287	64.39	0.7202	0.0034
91	flaA_2_870/1719	63.95	0.7153	0.0034
92	ttuB_870/1233	63.36	0.7087	0.0034
93	thiG_428/777	63.24	0.7073	0.0034
94	rimO_1063/1320	63.24	0.7073	0.0034
95	thiG_272/777	63.23	0.7072	0.0034
96	nrdA_1053/2370	63.23	0.7072	0.0034
97	nrdA_1047/2370	63.22	0.7071	0.0034
98	dksA_57/363	63.21	0.707	0.0034
99	rlmB_577/684	63.11	0.7059	0.0034
100	mnmG_1764/1860	63.08	0.7055	0.0034
101	arsB_276/1287	63.07	0.7054	0.0034
102	thiG_243/777	62.90	0.7035	0.0033
103	mnmG_1347/1860	62.83	0.7027	0.0033
104	mdtI_74/309	62.82	0.7027	0.0033
105	kdpC_363/411	62.81	0.7026	0.0033
106	kdpC_180/411	62.80	0.7025	0.0033
107	birA_402/654	62.80	0.7024	0.0033
108	kdsD_153/948	62.63	0.7005	0.0033
109	fmt_293/918	62.57	0.6999	0.0033
110	fmt_375/918	61.96	0.693	0.0033
111	lpxC_270/885	61.80	0.6912	0.0033
112	dapE_897/1098	61.66	0.6897	0.0033
113	argS_1197/1593	61.43	0.6871	0.0033
114	mnmG_1521/1860	61.42	0.687	0.0033
115	mnmG_1476/1860	61.42	0.6869	0.0033
116	mnmG_1455/1860	61.41	0.6868	0.0033
117	ackA_807/1191	61.40	0.6868	0.0033
118	mnmG_1437/1860	61.40	0.6868	0.0033

119	arsB_450/1287	61.39	0.6867	0.0033
120	ackA_825/1191	61.39	0.6866	0.0033
121	thiD_520/813	61.34	0.6861	0.0033
122	citN_821/1347	60.91	0.6813	0.0032
123	recN_540/1524	60.71	0.6791	0.0032
124	argD_625/1182	60.70	0.679	0.0032
125	parB_63/837	60.54	0.6771	0.0032
126	korA_822/1125	60.53	0.677	0.0032
127	recN_738/1524	60.52	0.6769	0.0032
128	prmC_345/816	60.38	0.6753	0.0032
129	nrdA_357/2370	60.37	0.6752	0.0032
130	htpX_974/1188	60.36	0.6751	0.0032
131	dltA_403/1509	60.35	0.675	0.0032
132	mnmG_669/1860	59.73	0.6681	0.0032
133	dapE_1065/1098	59.69	0.6677	0.0032
134	hemC_750/924	59.64	0.6671	0.0032
135	thiG_331/777	59.58	0.6664	0.0032
136	thiD_580/813	59.57	0.6663	0.0032
137	glgC_2_282/762	59.56	0.6662	0.0032
138	ackA_789/1191	59.50	0.6655	0.0032
139	arsB_796/1287	59.50	0.6655	0.0032
140	arsB_715/1287	59.49	0.6654	0.0032
141	arsB_616/1287	59.48	0.6653	0.0032
142	rcsC_2238/2310	59.11	0.6612	0.0031
143	pleD_81/1245	59.10	0.6611	0.0031
144	yrdA_405/549	59.10	0.661	0.0031
145	pleD_585/1245	59.10	0.661	0.0031
146	cydB_373/1125	59.09	0.6609	0.0031
147	recN_51/1524	59.09	0.6609	0.0031
148	infA_80/219	59.09	0.6609	0.0031
149	copA_1_1610/2352	59.08	0.6609	0.0031
150	infA_33/219	59.08	0.6608	0.0031
151	argH_1045/1383	59.08	0.6608	0.0031
152	ftsA_501/1383	59.07	0.6607	0.0031
153	soj_435/786	58.97	0.6596	0.0031
154	soj_123/786	58.97	0.6596	0.0031
155	soj_351/786	58.96	0.6595	0.0031
156	queE_385/744	58.96	0.6595	0.0031
157	soj_325/786	58.96	0.6594	0.0031
158	porA_187/1275	58.95	0.6594	0.0031

159	birA_603/654	58.95	0.6594	0.0031
160	soj_144/786	58.95	0.6593	0.0031
161	mntB_678/804	58.95	0.6593	0.0031
162	atpC_183/390	58.94	0.6593	0.0031
163	fmt_867/918	58.94	0.6592	0.0031
164	aspS_250/1752	58.94	0.6592	0.0031
165	fabF_189/1215	58.93	0.6591	0.0031
166	aat_2116/2130	58.93	0.6591	0.0031
167	aspS_706/1752	58.83	0.658	0.0031
168	aspS_687/1752	58.82	0.6579	0.0031
169	soj_387/786	58.81	0.6578	0.0031
170	sucD_606/870	58.71	0.6567	0.0031
171	sucD_537/870	58.70	0.6566	0.0031
172	sucD_624/870	58.70	0.6565	0.0031
173	pal_20/498	58.69	0.6565	0.0031
174	sucD_613/870	58.69	0.6564	0.0031
175	mutS2_378/2208	58.69	0.6564	0.0031
176	lpxC_633/885	58.68	0.6563	0.0031
177	recN_1225/1524	58.58	0.6552	0.0031
178	dcuB_136/1425	58.50	0.6544	0.0031
179	sucD_408/870	58.50	0.6543	0.0031
180	dcuB_102/1425	58.50	0.6543	0.0031
181	rpoN_565/1251	58.49	0.6542	0.0031
182	clpB_675/2574	58.49	0.6542	0.0031
183	flaA_2_742/1719	58.48	0.6541	0.0031
184	flaA_2_723/1719	58.42	0.6534	0.0031
185	rlmB_276/684	58.19	0.6509	0.0031
186	feoB_1098/1842	58.18	0.6508	0.0031
187	aer_1_65/498	58.15	0.6504	0.0031
188	aer_1_21/498	58.14	0.6503	0.0031
189	flaA_2_826/1719	57.92	0.6478	0.0031
190	rocA_2252/3489	57.72	0.6456	0.0031
191	uvrB_189/1974	57.53	0.6434	0.0031
192	yycB_1045/1164	57.52	0.6433	0.0031
193	uvrB_1704/1974	57.52	0.6433	0.0031
194	yajR_935/1296	57.51	0.6433	0.0031
195	uvrB_1513/1974	57.51	0.6432	0.0031
196	tolB_212/1209	57.51	0.6432	0.0031
197	uvrB_943/1974	57.50	0.6432	0.0031
198	uvrB_120/1974	57.50	0.6431	0.0031

199	tolB_132/1209	57.50	0.6431	0.0031
200	uvrB_1089/1974	57.49	0.6431	0.0031
201	sucC_48/1164	57.49	0.643	0.0031
202	rpoBC_2_3641/4554	57.49	0.643	0.0031
203	ttcA_105/756	57.49	0.643	0.0031
204	sucC_237/1164	57.48	0.6429	0.0031
205	rhaM_131/318	57.48	0.6429	0.0031
206	rpsA_110/1671	57.48	0.6429	0.0031
207	proP_4_560/1296	57.47	0.6428	0.0031
208	moaA_201/963	57.47	0.6428	0.0031
209	rpoBC_2_3850/4554	57.47	0.6428	0.0031
210	proP_2_1116/1362	57.47	0.6428	0.0031
211	mnmA_1_845/1017	57.46	0.6427	0.0031
212	plsY_460/609	57.46	0.6427	0.0031
213	mdh_633/903	57.46	0.6426	0.0031
214	kdpD_30/1821	57.45	0.6426	0.0031
215	murD_1080/1209	57.45	0.6426	0.0031
216	kdpD_702/1821	57.45	0.6426	0.0031
217	kdpD_1798/1821	57.45	0.6425	0.0031
218	kdpD_559/1821	57.44	0.6425	0.0031
219	kdpB_948/2046	57.44	0.6424	0.0031
220	icd2_390/2205	57.44	0.6424	0.0031
221	kdpD_4/1821	57.43	0.6424	0.0031
222	kdpB_1874/2046	57.43	0.6424	0.0031
223	htpX_655/1188	57.43	0.6423	0.0031
224	kdpB_1391/2046	57.42	0.6423	0.0031
225	htpX_143/1188	57.42	0.6422	0.0031
226	frdC_727/783	57.42	0.6422	0.0031
227	icd2_700/2205	57.42	0.6422	0.0031
228	hisS_1012/1227	57.41	0.6422	0.0031
229	ffh_428/1338	57.41	0.6421	0.0031
230	gpmI_506/1479	57.41	0.6421	0.0031
231	dsbL_1_78/360	57.40	0.6421	0.0031
232	btuD_1_265/855	57.40	0.642	0.0031
233	fusA_1331/2076	57.40	0.642	0.0031
234	dnaE_3535/3603	57.40	0.642	0.0031
235	atpC_290/390	57.39	0.6419	0.0031
236	copA_1_1242/2352	57.39	0.6419	0.0031
237	atpC_253/390	57.39	0.6419	0.0031
238	clpA_1936/2130	57.38	0.6418	0.0031

239	argC_58/1029	57.38	0.6418	0.0031
240	ccpA_24/1026	57.38	0.6418	0.0031
241	atoC_1248/1302	57.38	0.6418	0.0031
242	aas_3474/3513	57.38	0.6417	0.0031
243	aroA_66/1287	57.37	0.6417	0.0031
244	aroA_36/1287	57.36	0.6416	0.0031
245	uvrB_1866/1974	57.20	0.6398	0.003
246	murC_1290/1299	57.20	0.6397	0.003
247	lpxC_483/885	57.19	0.6396	0.003
248	kdpB_674/2046	57.19	0.6396	0.003
249	kdpD_615/1821	57.18	0.6395	0.003
250	kdpB_1818/2046	57.18	0.6395	0.003
251	kdpD_1593/1821	57.17	0.6395	0.003
252	kdpB_1614/2046	57.17	0.6394	0.003
253	kdpB_1063/2046	57.17	0.6394	0.003
254	kdpB_723/2046	57.16	0.6394	0.003
255	kdpB_1494/2046	57.16	0.6393	0.003
256	htpX_399/1188	57.16	0.6393	0.003
257	kdpB_1453/2046	57.15	0.6392	0.003
258	kdpB_1139/2046	57.14	0.6392	0.003
259	tmk_380/579	57.10	0.6386	0.003
260	dcuB_24/1425	57.09	0.6385	0.003
261	pfs_528/687	57.04	0.638	0.003
262	kdpA_1_179/261	57.03	0.6379	0.003
263	eptA_1119/1539	56.94	0.6369	0.003
264	cfiB_237/1446	56.90	0.6364	0.003
265	thiG_474/777	56.86	0.636	0.003
266	mnmA_1_426/1017	56.86	0.636	0.003
267	flaA_2_855/1719	56.45	0.6313	0.003
268	flaA_2_819/1719	56.44	0.6313	0.003
269	aer_1_234/498	56.43	0.6312	0.003
270	ackA_456/1191	56.09	0.6273	0.003
271	yafP_79/459	55.94	0.6257	0.003
272	flaA_2_1554/1719	55.93	0.6256	0.003
273	dapA_1_162/909	55.92	0.6255	0.003
274	atpD_1_437/522	55.92	0.6254	0.003
275	thiG_171/777	55.86	0.6248	0.003
276	smpB_189/453	55.85	0.6247	0.003
277	yflS_134/168	55.85	0.6246	0.003
278	rpoBC_1_4034/4128	55.84	0.6246	0.003

279	gppA_2_197/975	55.84	0.6246	0.003
280	tsf_246/1074	55.84	0.6246	0.003
281	kgtP_424/1260	55.84	0.6245	0.003
282	glyS_1573/1995	55.84	0.6245	0.003
283	hom_842/1248	55.83	0.6244	0.003
284	glmU_27/1290	55.83	0.6244	0.003
285	aas_2194/3513	55.83	0.6244	0.003
286	hom_589/1248	55.82	0.6244	0.003
287	dapE_717/1098	55.82	0.6243	0.003
288	aas_1587/3513	55.82	0.6243	0.003
289	carB_1236/3270	55.81	0.6243	0.003
290	bioB_183/837	55.80	0.6242	0.003
291	mog_477/543	55.78	0.6239	0.003
292	copA_1_837/2352	55.78	0.6239	0.003
293	copA_1_783/2352	55.77	0.6238	0.003
294	bamA_294/2220	55.77	0.6237	0.003
295	copA_1_1335/2352	55.76	0.6237	0.003
296	bamA_279/2220	55.76	0.6237	0.003
297	clpA_1341/2130	55.75	0.6236	0.003
298	atpC_102/390	55.75	0.6236	0.003
299	birA_348/654	55.74	0.6235	0.003
300	asd_867/1032	55.74	0.6235	0.003

Table S2. Metadata for extraintestinal *Campylobacter jejuni.*

Strain Name	Host	Disease	Sources	Region	Year	Clone SA*	ST	Accession
CA6e	Sheep	Abortion	Aborted placenta	CA,USA	1991	Yes	8	SRR3094400
CA7e	Sheep	Abortion	Aborted placenta	CA,USA	1992	Yes	8	SRR3094401
ID1	Sheep	Abortion	Aborted placenta	ID,USA	1992	Yes	8	SRR3094402
CA8e	Sheep	Abortion	Aborted placenta	CA,USA	1993	Yes	8	SRR3094403
ID12	Sheep	Abortion	Aborted placenta	ID,USA	1993	Yes	8	SRR3094404
ID15	Sheep	Abortion	Aborted placenta	ID,USA	1993	Yes	8	SRR3094405
ID28	Sheep	Abortion	Aborted placenta	ID,USA	1993	Yes	8	SRR3094406
ID31	Sheep	Abortion	Aborted placenta	ID,USA	1993	Yes	8	SRR3094407
ID34	Sheep	Abortion	Aborted placenta	ID,USA	1993	Yes	8	SRR3094408
CA3e	Sheep	Abortion	Aborted placenta	CA,USA	1999	Yes	8	SRR3094409
CA4e	Sheep	Abortion	Aborted placenta	CA,USA	1999	Yes	8	SRR3094410
CA5e	Sheep	Abortion	Aborted placenta	CA,USA	2000	Yes	8	SRR3094411
VDL705	Sheep	Abortion	Aborted placenta	IA,USA	2003	Yes	8	SRR3094412
VLD698	Sheep	Abortion	Aborted placenta	IA,USA	2003	Yes	8	SRR3094413
CA7	Sheep	Abortion	Aborted placenta	CA,USA	2003	Yes	8	SRR3094414
ID181077-B	Sheep	Abortion	Aborted placenta	ID,USA	2004	Yes	8	SRR3094415
ID323147-F	Sheep	Abortion	Aborted placenta	ID,USA	2004	Yes	8	SRR3094416
VDL5908	Sheep	Abortion	Aborted placenta	IA,USA	2004	Yes	8	SRR3094417
VDL3842	Sheep	Abortion	Aborted placenta	IA,USA	2004	Yes	8	SRR3094418
CA5	Sheep	Abortion	Aborted placenta	CA,USA	2004	Yes	8	SRR3094419
CA8	Sheep	Abortion	Aborted placenta	CA,USA	2004	Yes	8	SRR3094420
ID219083-A	Sheep	Abortion	Aborted placenta	ID,USA	2005	Yes	8	SRR3094421
VDL3080	Sheep	Abortion	Aborted placenta	IA,USA	2005	Yes	8	SRR3094422
SD3831	Sheep	Abortion	Aborted placenta	SD,USA	2005	Yes	8	SRR3094423
CA3	Sheep	Abortion	Aborted placenta	CA,USA	2005	Yes	8	SRR3094424
ID273138-G	Sheep	Abortion	Aborted placenta	ID,USA	2006	Yes	8	SRR3094425

ID428211-A	Sheep	Abortion	Aborted placenta	ID,USA	2006	Yes	8	SRR3094426
ID147052-A	Sheep	Abortion	Aborted placenta	ID,USA	2007	Yes	8	SRR3094427
VDL6220	Sheep	Abortion	Aborted placenta	IA,USA	2007	Yes	8	SRR3094428
ND6	Sheep	Abortion	Aborted placenta	ND,USA	2007	Yes	8	SRR3094429
VDL2192	Sheep	Abortion	Aborted placenta	IA,USA	2008	Yes	8	SRR3094430
VDL8958	Sheep	Abortion	Aborted placenta	IA,USA	2009	Yes	8	SRR3094431
VDL35044	Sheep	Abortion	Aborted placenta	IA,USA	2009	Yes	8	SRR3094432
VDL1957	Sheep	Abortion	Aborted placenta	IA,USA	2010	Yes	8	SRR3094433
VDL2764	Sheep	Abortion	Aborted placenta	IA,USA	2010	Yes	8	SRR3094434
VDL5414	Sheep	Abortion	Aborted placenta	IA,USA	2011	Yes	8	SRR3094435
CO351	Sheep	Abortion	Aborted placenta	CO,USA	2011	Yes	8	SRR3094436
ID33	Sheep	Abortion	Aborted placenta	ID,USA	1993	No	441	SRR3094437
CA2	Sheep	Abortion	Aborted placenta	CA,USA	2003	No	607	SRR3094444
VDL4646	Sheep	Abortion	Aborted placenta	IA,USA	2004	No	806	SRR3094445
ND7	Sheep	Abortion	Aborted placenta	ND,USA	2007	No	42	SRR3094447
ND9	Sheep	Abortion	Aborted placenta	ND,USA	2008	Yes	8	SRR3094448
VDL902	Sheep	Abortion	Aborted placenta	IA,USA	2008	No	982	SRR3094449
VDL2019	Sheep	Abortion	Aborted placenta	IA,USA	2008	No	5189	SRR3094450
VDL213	Sheep	Abortion	Aborted placenta	IA,USA	2009	No	806	SRR3094451
VDL6069	Sheep	Abortion	Aborted placenta	IA,USA	2010	No	38	SRR3094453
CA10	Cattle	Abortion	Aborted placenta	CA,USA	2003	Yes	8	SRR3094454
CA12	Cattle	Abortion	Aborted placenta	CA,USA	2003	Yes	8	SRR3094455
CA11	Cattle	Abortion	Aborted placenta	CA,USA	2004	Yes	8	SRR3094456
ND3	Cattle	Abortion	Aborted placenta	ND,USA	2005	Yes	8	SRR3094457
VDL31248	Cattle	Abortion	Aborted placenta	IA,USA	2009	Yes	8	SRR3094458
VDL2738	Goat	Abortion	Aborted placenta	IA,USA	2005	Yes	8	SRR3094459
VDL1625	Goat	Abortion	Aborted placenta	IA,USA	2008	Yes	8	SRR3094460
VDL4023	Goat	Abortion	Aborted placenta	IA,USA	2010	Yes	8	SRR3094461

VDL4350	Goat	Abortion	Aborted placenta	IA,USA	2010	Yes	8	SRR3094462
1E2B2a	Sheep		Bile	IA,USA	2008	Yes	8	SRR3094463
1L1F1a	Sheep		Feces	IA,USA	2008	Yes	8	SRR3094464
2L5B2	Sheep		Bile	IA,USA	2008	Yes	8	SRR3094465
2L5B4	Sheep		Bile	IA,USA	2008	Yes	8	SRR3094466
D6249	Human	Gastroenteritis	Feces	VT,USA	2003	Yes	8	SRR3094467
D7324	Human	Gastroenteritis	Feces	PA,USA	2008	Yes	8	SRR3094469
D7365	Human	Gastroenteritis	Feces	RI,USA	2008	Yes	8	SRR3094470
D8347	Human	Gastroenteritis	Feces	MT,USA	2010	Yes	8	SRR3094471
FDA17848	Human	Gastroenteritis	Feces	IA,USA	Unknown	Yes	8	SRR3094472
FDA17817	Human	Gastroenteritis	Feces	IA,USA	Unknown	Yes	8	SRR3094473
FDAN342	Chicken		Meat	MN,USA	2004	Yes	8	SRR3094475
UK33	Sheep	Abortion	Aborted placenta	UK	2006	No	227	SRR3094476
UK29	Sheep	Abortion	Aborted placenta	UK	2005	No	21	SRR3094477
OF48	Sheep		Feces	IA,USA	2006	No	61	SRR3094479
VDL35490	Sheep	Abortion	Aborted placenta	IA,USA	2012	No	982	SRR3094480
VDL3452	Sheep	Abortion	Aborted placenta	IA,USA	2013	Yes	8	SRR3094481
VDL6794	Sheep	Abortion	Aborted placenta	IA,USA	2013	Yes	8	SRR3094482
UK40	Sheep	Abortion	Aborted placenta	UK	2008	No	227	SRR3094483
UK10	Sheep	Abortion	Aborted placenta	UK	2003	No	206	SRR3094485
UK32	Sheep	Abortion	Aborted placenta	UK	2006	No	42	SRR3094487
UK37	Sheep	Abortion	Aborted placenta	UK	2007	No	270	SRR3094488
ID7	Sheep	Abortion	Aborted placenta	ID,USA	1992	Yes	8	SRR3094489
ID27	Sheep	Abortion	Aborted placenta	ID,USA	1993	Yes	8	SRR3094490
ID25	Sheep	Abortion	Aborted placenta	ID,USA	1993	Yes	21	SRR3094491
SD4165	Sheep	Abortion	Aborted placenta	SD,USA	2005	Yes	8	SRR3094492
VDL2847	Sheep	Abortion	Aborted placenta	IA,USA	2010	Yes	8	SRR3094493
VDL2918	Sheep	Abortion	Aborted placenta	IA,USA	2005	Yes	8	SRR3094494

2L5F9	Sheep		Feces	IA,USA	2008	Yes	8	SRR3094495
302	Sheep	Abortion	Aborted placenta	IA,USA	2014	Yes	8	SRR3094496
3008	Sheep	Abortion	Aborted placenta	IA,USA	2014	Yes	8	SRR3094497
5406	Sheep	Abortion	Aborted placenta	MI,USA	2014	Yes	8	SRR3094498

Table S3. Metadata for intestinal *Campylobacter jejuni.*

Genome	Collection Date	Geographic location
197.14907	2018	USA:PA
197.15041	2018	USA:MN
197.15927	2016	USA:TX
197.15936	2016	USA:PA
197.15943	2016	USA:TN
197.15952	2016	USA:PA
197.15957	2016	USA:OR
197.15958	2016	USA:NE
197.15964	2016	USA:ID
197.15966	2016	USA:CO
197.15972	2016	USA:NJ
197.15982	2016	USA:TX
197.15983	2016	USA:PA
197.15988	2016	USA:MI
197.15993	2016	USA:PA
197.15994	2016	USA:MI
197.15997	2016	USA:NY
197.15999	2016	USA:OH
197.16001	2016	USA:GA
197.16005	2016	USA:OH
197.16021	2016	USA:PA
197.16023	2016	USA:PA
197.16031	2016	USA:WI
197.16033	2016	USA:CA
197.16041	2016	USA:TX
197.16042	2016	USA:NE
197.16048	2016	USA:TX
197.16051	2016	USA:WA
197.16053	2016	USA:MI
197.16058	2016	USA:NJ
197.16060	2016	USA:WA
197.16064	2016	USA:NE
197.16072	2016	USA:MI
197.16074	2016	USA:ID
197.16075	2016	USA:MI
197.16078	2016	USA:CA
197.16079	2016	USA:OR
197.16084	2016	USA:FL
197.16099	2016	USA:NJ
197.16103	2016	USA:TX

197.16118	2016	USA:WI
197.16127	2016	USA:MN

Table S4. Confusion matrix and derived model metrics for the XGboost model with extraintestinal *Campylobacter jejuni*. TP= True positive, FN= False Negative, FP= False Positive, FN= False Negative.

	**Extraintestinal**	**Intestinal**
**Extraintestinal**	**18358**	**389964**
**Intestinal**	**6003**	**821898**

**Measure**	**Value**	**Formula**
Sensitivity	0.7536	TP / (TP + FN)
Specificity	0.6782	TN / (FP + TN)
Precision	0.045	TP / (TP + FP)
Negative Predictive Value	0.9927	TN / (TN + FN)
False Positive Rate	0.3218	FP / (FP + TN)
False Discovery Rate	0.955	FP / (FP + TP)
False Negative Rate	0.2464	FN / (FN + TP)
Accuracy	0.6797	(TP + TN) / (P + N)
F1 Score	0.0849	2TP / (2TP + FP + FN)
Matthews Correlation Coefficient	0.1276	TP*TN - FP*FN / sqrt((TP+F P)*(TP+FN)*(TN+FP)*(TN+FN))

Sensitivity Specificity Precision
Negative Predictive Value False Positive Rate
False Discovery Rate False Negative Rate
Accuracy
F1 Score
Matthews Correlation Coefficient
